# Engineering Zn/Fe Mixed Metal Oxides with Tunable Structural and Magnetic Properties for Magnetic Particle Imaging

**DOI:** 10.3390/nano14231964

**Published:** 2024-12-07

**Authors:** Qianyi Zhang, Bing Sun, Saeed Shanehsazzadeh, Andre Bongers, Zi Gu

**Affiliations:** 1 School of Chemical Engineering, University of New South Wales, Sydney, NSW 2052, Australia; qianyi.zhang@sydney.edu.au (Q.Z.); bing.sun@uq.net.au (B.S.); 2 School of Chemistry, University of Sydney, Sydney, NSW 2006, Australia; 3 Biological Resources Imaging Laboratory, Mark Wainwright Analytical Centre, The University of New South Wales, Sydney, NSW 2052, Australia; s.shanehsazzadeh@unsw.edu.au (S.S.); andre.bongers@unsw.edu.au (A.B.); 4 Australian Centre for NanoMedicine (ACN), University of New South Wales, Sydney, NSW 2052, Australia; 5 UNSW RNA Institute, University of New South Wales, Sydney, NSW 2052, Australia

**Keywords:** magnetic particle imaging, metal oxide nanoparticles, layered double hydroxide, structure–function relationship

## Abstract

Engineering magnetic nanoparticles with tunable structural properties and magnetism is critical to develop desirable magnetic particle imaging (MPI) tracers for biomedical applications. Here we present a new superparamagnetic metal oxide nanoparticle with a controllable chemical composition and magnetism for imaging tumor xenografts in living mice. Superparamagnetic Zn/Fe mixed metal oxide (ZnFe-MMO) nanoparticles are fabricated via a facile one-pot co-precipitation method in water followed by thermal decomposition with tunable Zn/Fe ratios and at various calcination temperatures. This work, for the first time, presented LDH-derived metal oxides for an MPI application. The metal composition is tunable to present an optimized MPI performance. The analytical results demonstrate that ZnFe-MMO nanoparticles at the designed molar ratio of Zn/Fe = 2:1 after 650 °C calcination demonstrate a higher saturation magnetization (M_S_) value and optimal MPI signal than the samples presented with other conditions. The excellent biocompatibility of ZnFe-MMO is demonstrated in both breast cancer cells and fibroblast cell cultures. In vivo imaging of 4T1 tumor xenografts in mice using ZnFe-MMO as a tracer showed that the mean signal intensity is 1.27-fold higher than the commercial tracer VivoTrax at 72 h post-injection, indicating ZnFe-MMO’s promise for prolonged MPI imaging applications.

## 1. Introduction

Magnetic particle imaging (MPI) is an emerging technology that has great potential as a new tracer imaging method. Currently being developed under pre-clinical conditions, it has strong merit for clinical translation [1]. The MPI concept was first proposed in 2005, which is a cutting-edge imaging technique associated with the saturation properties of magnetic materials [2]. This technique has attracted widespread attention because of its ability to localize and quantify small tracer amounts with high sensitivity and without tissue backgrounds in living subjects. Compared to classical tracking methods, it has unique advantages including being radiation-free, deep tissue penetration, and linear quantification [3].

MPI contrast is created directly by the magnetic nanoparticle tracers under a time-varying magnetic field, which results in an MPI intensity that is directly proportional to the iron mass of the injected tracers [4]. A vital property of MPI contrast is the magnetic moment, which is proportional to the saturation magnetization (M_S_) of the constituting material [5]. To obtain optimal signal intensity, MPI tracers should be superparamagnetic with an improved M_S_ value [6]. Currently, standard commercial MPI tracers are repurposed SPION MRI contrast agents (VivoTrax and Resovist), originally used for T_2_-MRI contrast. Albeit their common use in MPI, these agents are optimized for MRI technology and are considered suboptimal for MPI [7]. Hence, many recent research efforts have focused on engineering magnetic particles to meet MPI requirements [8,9,10,11,12]. Some recently established synthetic methods care about the magnetic core composition, size, and shape of particles, which include stringent organic solvents as surfactants in synthetic processes [13,14]. Interestingly, transition metal ion (e.g., Zn, Mn, Co, and Ni) doping has been found to feasibly introduce disorder and impurity into the lattice structure to improve the total M_S_ value of nanoparticles for MPI [15,16]. Metal doping increases the overall anisotropy of the nanoparticle and partially alleviates the antiparallel exchange coupling between Fe^3+^, which increases the M_S_ value of magnetic nanoparticles [15,17]. Although metal doping is a promising approach, precise synthesis conditions and their influence on physicochemical properties, magnetic properties, and MPI signals are not fully understood.

Layered double hydroxides (LDHs) can feasibly be synthesized in water and represent an ideal metal reservoir for many biomedical applications [18,19,20,21]. Mixed metal oxide (MMO) nanoparticles derived from the thermal decomposition of LDH are constituted by a metal oxide phase and spinel-like phase, showing higher magnetic properties than those fabricated by conventional ceramic or wet chemical routes that require organic solvents [22]. The decomposition procedure of LDH has revealed that the nucleation of the metal oxide phase occurs at temperatures below 300 °C, while the spinel phase can be gradually observed in a temperature range of 500–800 °C [23].

Herein, we synthesized a series of ZnFe-MMO nanoparticles by the thermal decomposition of LDH prepared in water with various Zn/Fe ratios at different calcination temperatures to investigate their effects on magnetic properties and MPI function. It is the first time that LDH-derived metal oxides were reported with a tunable metal composition to present an optimized MPI performance. Firstly, the physiochemical properties of MMO nanoparticles were characterized, including the particle size, morphology, chemical composition, and crystallinity. The saturation magnetization values were measured via magnetic hysteresis. The two-dimensional projection of MPI and a line MPI spectrum of ZnFe-MMO nanoparticles, together with MPI sensitivity and resolution, were measured on an MPI scanner. After confirming the biocompatibility of the ZnFe-MMO nanoparticles using two cell lines, the MPI performance was assessed in a 4T1 breast cancer xenografted mouse model, using the commercial MPI tracer as a reference control.

## 2. Materials and Methods

### 2.1. Materials

Iron(III) chloride (FeCl_3_, >97%), Zinc(II) chloride (ZnCl_2_, >95%), and NaOH (>98%) were purchased from Sigma-Aldrich (Sydney, NSW, AU). Dimethyl Sulfoxide (DMSO, sterile) was purchased from cell signaling. Roswell Park Memorial Institute medium (RPMI 1640) and penicillin/streptomycin were bought from Gibco (Sydney, NSW, Australia). Fetal bovine serum (FBS) and trypsin-EDTA (0.25%, phenol red) were purchased from Life Technologies Australia (Sydney, NSW, AU). Cell counting kit-8 (CCK-8) was bought from Abcam (Melbourne, VIC, AU). VivoTrax Plus was purchased from Magnetic Insight (Alameda, CA, USA).

### 2.2. Synthesis of ZnFe-MMO Nanoparticles in Different Zn/Fe Ratios

The co-precipitation method was employed to synthesize ZnFe-LDH with a Zn/Fe molar ratio of 2:1, 3:1, and 4:1. A typical method to produce Zn_3_Fe-LDH is as follows: Solution A (5 mL) containing ZnCl_2_ (3 mmol) and FeCl_3_ (1 mmol) was mixed with Solution B (5 mL) containing NaOH (8 mmol) with a constant pH value. The mixture then underwent vigorous stirring at room temperature for 20 h. The Zn_3_Fe-LDH nanoparticles were collected by centrifugation twice at 5000× *g* for 5 min.

After removing extra salt, the nanoparticle pallet in different Zn/Fe ratios was dried to a powder, followed by single-step calcination at 650 °C for 3 h under N_2_ protection to obtain Zn_2_Fe-MMO-650, Zn_3_Fe-MMO-650, and Zn_4_Fe-MMO-650 nanoparticles.

### 2.3. Synthesis of Zn_2_Fe-MMO in Different Calcination Temperatures

The Zn_2_Fe-LDH precursor was prepared by adopting the aforementioned procedure but feeding the Zn/Fe molar ratio to 2:1. The Zn_2_Fe-LDH nanoparticles were collected by centrifugation twice at 5000× *g* for 5 min. After removing extra salt, the nanoparticle pallet was dried to a powder, followed by single-step calcination at 550, 750, and 850 °C for 3 h, respectively, to obtain Zn_2_Fe-MMO-550, Zn_2_Fe-MMO-750, and Zn_2_Fe-MMO-850 nanoparticles.

### 2.4. Measurement of Saturation Magnetization

The M-H loops of ZnFe-MMO nanoparticles were obtained by fixing the nanoparticle powders in a plastic tube at an iron amount of 3 mg in a superconducting quantum interference device (SQUID) on a Quantum Design MPMS XL (Quantum Design Inc., San Diego, CA, USA) magnetometer. To ensure the accuracy of magnetic diameter fits, magnetization data were obtained over a large enough range of −70 kOe to 70 kOe under 300 K to fully saturate the particles in multiple steps. The field steps are not the same interval, depending on the magnitude of the field applied. At a higher field (>|1| T), larger steps were used, and smaller steps for a lower field (e.g., 7T to 1T @ 1T step, 0.8T to 0.4T @0.2T step, 0.3T to 0.05T @ 0.05T step, and then 0.01T step down to 0T).

### 2.5. Cell Culture

The breast cancer cells 4T1 and fibroblast cells NIH/3T3 were cultured in a growth medium (RPMI1640 with glutamine) supplemented with 10% fetal bovine serum (FBS), streptomycin (100 mg mL^−1^), and penicillin (100 units mL^−1^). The cells were cultured at 37 °C in a humidified atmosphere with 5% CO_2_ in air.

### 2.6. Cytotoxicity Study of Zn_2_Fe-MMO-650 Nanoparticles

The 4T1 cells and NIH/3T3 cells were seeded (1 × 10^4^ cells in 100 μL of RMPI1640 and DMEM per well) in 96-well microplates and allowed to adhere overnight. The culture medium was then replaced with fresh RPMI and DMEM containing Zn_2_Fe-MMO-650 at Fe concentrations of 0, 0.05, 0.25, 5, 10, 100, and 200 μg mL^−1^. After 24 h of incubation, the culture media was replaced with RPMI and DMEM media containing 10% CCK-8. After 1 h of incubation, cell viability was determined by comparing the absorbance at λ = 450 nm to the control group. The absorbance of samples was measured on a microplate reader (FLUOstar, Omega, Melbourne, VIC, AU ). The experiments were carried out in duplicate, and the values from each experiment were calculated from 3 wells.

### 2.7. Tumor Model

All experiments on animals were performed with the approval of the University of New South Wales Laboratory Animal Centre Ethics Committee. BALB/c female white mice (20 ± 2 g) were subcutaneously injected with 4T1 breast cancer cells (200 μL, 2 × 10^6^) on their left rumps to develop the subcutaneous tumor model. The tumor implantation site was shaved before injection, and the MPI tracker was injected when the tumor volume reached 100 mm^3^. The length (L) and width (W) of the tumors were measured by a caliper, and the tumor volume (V) was calculated with the formulation V (mm^3^) = 0.5 × L × W^2^ every day after tumor inoculation.

### 2.8. Magnetic Particle Imaging Evaluation

The magnetic particle imaging (MPI) performance of the nanoparticles (NPs) was evaluated through a 2D projection scan using the MOMENTUM pre-clinical scanner from Magnetic Insight, Inc. Each sample underwent individual scanning using the RELAX™ module on the MOMENTUM™ imager, generating a point spread function (PSF) for each. The imaging process involved acquiring 2D images with a 5.7 T/m selection field gradient and excitation field strengths of 20 mT and 26 mT in the X and Z channels, respectively. For in vitro tests, we employed the standard mode, and for in vivo experiments, we utilized the high sensitivity mode. Throughout the MPI scanning, all samples maintained a constant frequency of 45 kHz [24].

These 2D images, covering a field of view (FOV) of 12 × 6 cm, took approximately 2.5 min to acquire. To prepare the nanoparticle solution, a stock solution with a concentration of 0.1 mg/mL of Fe was created, and the concentration was quantified using Inductively Coupled Plasma Optical Emission Spectrometry (ICP-OES) (Santa Clara, CA, USA). Using the 0.1 mg Fe mL^−1^ stock solution, five different concentrations were formulated, ranging from 5 μg mL^−1^ to 100 μg mL^−1^. The MPI measurements were conducted on both VivoTrax and Zn_2_Fe-MMO-650.

During the in vivo experiments, mice bearing 4T1 subcutaneous tumors were divided into two groups (n = 3 mice). One group received an i.t. injection of Vivotrax (40 μL, 145 μg Fe mL^−1^) into the tumor. The second group was i.t. injected with Zn_2_Fe-MMO-650 (40 μL, 145 μg Fe mL^−1^). MP images were acquired at 24 and 72 h post-injection. The mice were anesthetized with 2% isoflurane at 2 L/min of oxygen flow delivered via a nose cone during the imaging sessions. A dorsal photo of the animal was taken before the MPI scanning using the inbuilt camera to provide a body shape reference for later overlay.

### 2.9. Characterization

X-ray diffraction (XRD) measurements were performed on LDH and MMO powder samples using a PANalytical Bragg–Brentano geometry X-ray diffractometer operated at 45 kV and 40 mA and fitted with a CoKα source. The wavelength was converted to CuKα using HighScore Plus software version 4.9. A scan rate of 0.01° min^−1^ was applied with a step size of *2θ* = 0.0260°. Fourier transform infrared spectroscopy (FTIR) was carried out on a Bruker IFS 66/S single-beam spectrometer (Billerica, MA, USA). Spectra were obtained at regular time intervals in the MIR region of 4000–400 cm^−1^ at a resolution of 4 cm^−1^ (64 scans). The ICP-MS data were collected from coupled plasma mass spectrometry. The morphology of nanoparticles was observed using a Transmission Electronic Microscope (TEM, FEI Tecnai G2, Hillsboro, OR, USA) at an accelerating voltage of 200 kV. X-ray photoelectron spectroscopy (XPS) was performed using a Thermo ESCALAB 250i spectrometer (Waltham, MA, USA) with a monochromatic X-ray source (AlKα, 1486.68 eV) operated at a 164 W emission power. XPS spectra were analyzed using Avantage 4.88 software. MPI measurements were conducted on an MPI scanner (Magnetic Insight Inc., MOMENTUM™ Imager, Alameda, CA, USA).

## 3. Results

### 3.1. Effect of Zn/Fe Molar Ratio on Physicochemical Properties, Magnetic Properties, and MPI Signal of ZnFe-MMO Nanoparticles

**Figure 1:**
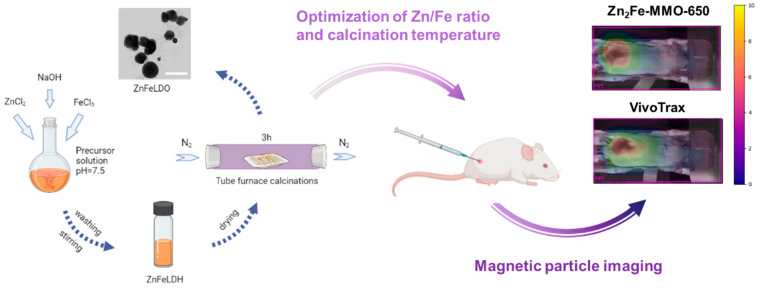
Schematic illustration of the synthesis and in vivo MPI evaluation of ZnFe-MMO nanoparticles. Created with BioRender.com.

**Figure 2:**
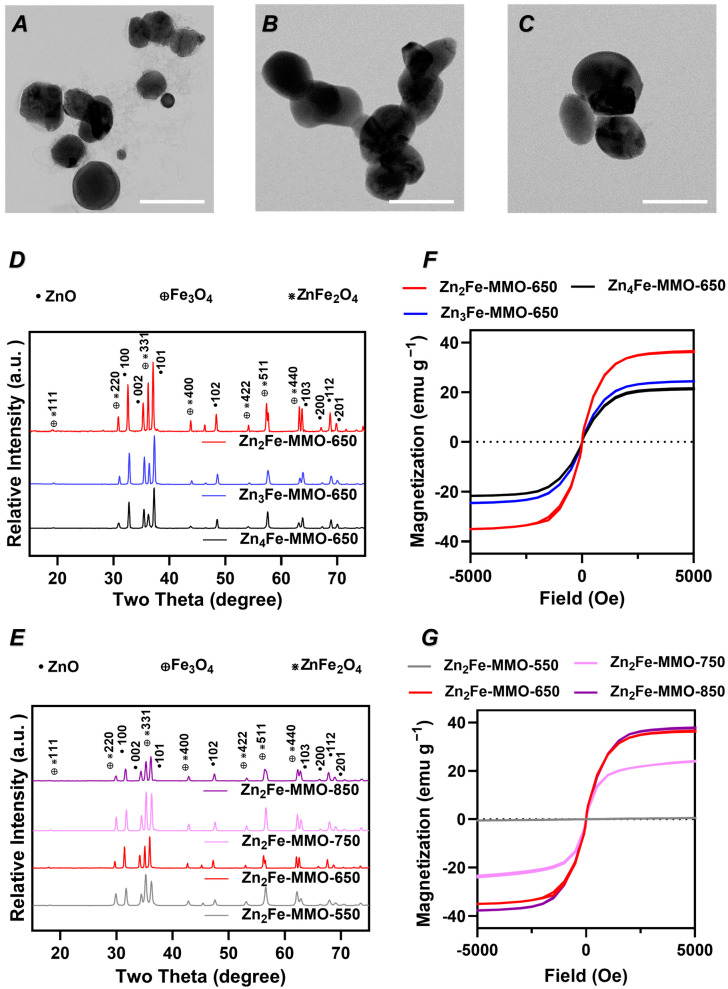
TEM images of (**A**) Zn_2_Fe-MMO-650, (**B**) Zn_3_Fe-MMO-650, and (**C**) Zn_4_Fe-MMO-650 (scale bar = 200 nm). PXRD patterns of (**D**) MMO in different Zn/Fe molar ratios and (**E**) MMO at different calcination temperatures. The saturation magnetization (M_S_) curve of MMO (**F**) in different Zn/Fe molar ratios and (**G**) at different calcination temperatures.

ZnFe-MMO nanoparticles were synthesized by the co-precipitation of metal cations followed by thermal decomposition (Figure 1). Firstly, Zn^2+^ and Fe^3+^ were co-precipitated with an alkaline solution to form ZnFe-containing layered double hydroxide (LDH) nanoparticles with different Zn/Fe molar ratios. The XRD patterns of all the LDHs showed typical basal reflections of lamellar LDH materials, as reflected by the (003), (006), (009), (012), (110), and (113) peaks (Figure S1). The (003) spacing of the LDH samples at designed Zn/Fe molar ratios of 2:1, 3:1, and 4:1 were calculated to be around 7.11, 7.04, and 7.02 nm, respectively, corresponding to the Cl-LDH in the literature [25]. The original ZnFe-LDH exhibited characteristic vibration peaks of LDH in which the typical vibration of the interlayer water molecule and the surface hydroxyl group were recorded at a wavenumber of 3484 cm^−1^ and 1636 cm^−1^ [26,27]. Moreover, the peaks observed in the range of 400–800 cm^−1^ are attributed to the lattice vibration of metal–oxygen–metal (M-O-M) bonding. The ZnFe-containing LDH materials were subsequently annealed under N_2_ conditions at different temperatures (550, 650, 750, and 850 °C) to obtain the ZnFe-MMO nanoparticles. The ZnFe-MMO with a designed Zn/Fe molar ratio of 2:1, 3:1, and 4:1 annealed at 650 °C (designated as Zn_2_Fe-MMO-650, Zn_3_Fe-MMO-650, and Zn_4_Fe-MMO-650, respectively) exhibited a quasi-spherical morphology and similar average particle size of 132 nm under TEM observation (Figure 2A–C). The ICP analysis of MMO samples showed that the Zn/Fe molar ratios in Zn_2_Fe-MMO-650, Zn_3_Fe-MMO-650, and Zn_4_Fe-MMO-650 were 1.5, 2.4, and 3.1, respectively, which were similar to the actual Zn/Fe molar ratios in the corresponding Zn_2_Fe-LDH, Zn_3_Fe-LDH, and Zn_4_Fe-LDH samples (Table S1). The actual Zn/Fe molar ratios were slightly lower than the designed molar ratios but maintained the increasing trend of doped Zn content in the MMO samples.

The crystallinity of the ZnFe-MMO nanoparticles was characterized via powder X-ray diffraction (PXRD) (Figure 2D). The diffraction peaks at (111), (220), (311), (400), (442), (551), and (440) corresponded to the crystalline planes of Fe_3_O_4_ and ZnFe_2_O_4_ [28]. The diffraction peaks at (100), (002), (101), (102), (103), (200), (112), and (201) corresponded with the typical hexagonal wurtzite structure of ZnO [29]. The above results revealed that the ZnFe-MMO nanoparticles were successfully synthesized. As shown in the Fourier transform infrared (FTIR) spectra of ZnFe-MMO, the vibration peaks corresponding to the interlayer water molecule and the interlayer anion have drastically decreased, in comparison to the FTIR spectra of the corresponding LDH materials (Figures S2 and S3). This finding indicated that the layered structure of the ZnFe-LDH collapsed due to the removal of the interlayer species as a result of calcination.

The magnetic hysteresis of ZnFe-MMO was measured on dried nanoparticle samples containing 3 mg of iron. The coercivity of all samples was not observed in the hysteresis loop (Figure 2F), indicating the superparamagnetic behavior of ZnFe-MMO nanoparticles. The M_S_ values of Zn_2_Fe-MMO-650, Zn_3_Fe-MMO-650, and Zn_4_Fe-MMO-650 were 22.8, 26.0, and 39.3 emu g^−1^, respectively, demonstrating the tunable magnetic properties of MMO nanoparticles based on varying metal molar ratios.

**Figure 3:**
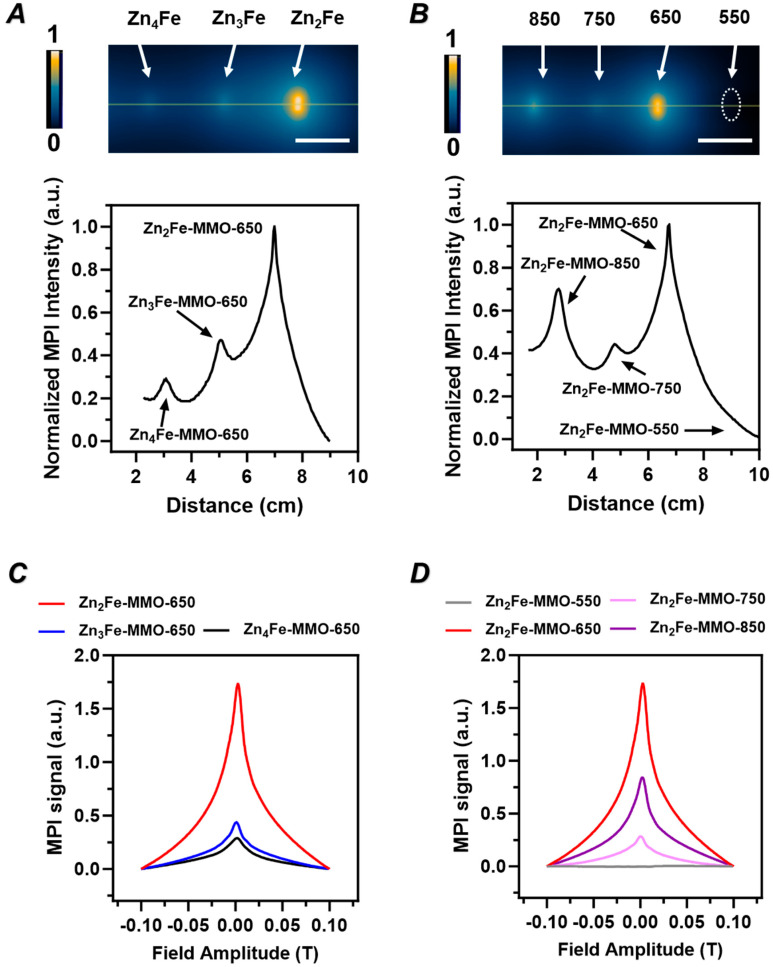
In vitro MPI performance of MMO in PCR tubes. Two-dimensional projection MPI images and corresponding intensities from Z channel (**A**) in different designed Zn/Fe molar ratios of MMO (scale bar = 2 cm) and (**B**) at different MMO calcination temperatures (50 µg of Fe). The dotted circle indicates Zn_2_Fe-MMO-550 sample location. (**C**,**D**) MPI point-of-spread function of the image (**A**,**B**).

An MPI evaluation of MMO nanoparticles containing different metal ratios was conducted on a MOMENTUM Imager. The comparison of projection signals in two-dimensional projections of MPI and the line MPI spectrum (Figure 3A) indicated that Zn_2_Fe-MMO-650 exhibited the strongest contrast ability. This is consistent with the MPI point-of-spread function obtained from the MPI oximeter measurements where Zn_2_Fe-MMO-650 also showed the most intensive MPI signal among all samples (Figure 3C). The MPI evaluation results correspond to the magnetic hysteresis of MMO nanoparticles, demonstrating that Zn_2_Fe-MMO-650 has the highest M_S_ value and MPI contrast. Therefore, the designed Zn/Fe molar ratio of 2:1 was selected to conduct the following studies.

### 3.2. Effect of Synthesis Temperature on Physicochemical Properties, Magnetic Properties, and MPI Signal of ZnFe-MMO

To investigate the effect of synthesis temperature, three ZnFe-MMO samples at the designed Zn/Fe molar ratio of 2:1 were synthesized by increasing the calcination temperature from 550 to 850 °C. The resultant samples were designated as Zn_2_Fe-MMO-550, Zn_2_Fe-MMO-750, and Zn_2_Fe-MMO-850, respectively. The particle size and morphology were observed under TEM. It is found that the Zn_2_Fe-MMO-550 samples showed a cubic shape with an average size of ca. 25 nm under the 550 °C treatment (Figure S4A). The particle size became larger when the calcination temperature increased from 550 to 850 °C. The size and morphology of Zn_2_Fe-MMO-750 samples appeared less uniform than the other samples (Figure S4B) while the Zn_2_Fe-MMO-850 samples exhibited an aggregated plate-like structure (Figure S4C). The PXRD patterns of four Zn_2_Fe-MMO nanoparticles synthesized at different calcination temperatures showed typical peaks of the metal oxide phase and spinel-like phase (Figure 2E). The FTIR spectra demonstrated the collapse of the LDH structure during thermal decomposition, confirming the successful fabrication of MMO nanoparticles (Figures S2 and S5). The magnetization curves of Zn_2_Fe-MMO are shown in Figure 2G, indicating that the synthesized MMO at 550 °C was not superparamagnetic since the magnetic strength increased linearly with the applied field. On the other hand, Zn_2_Fe-MMO treated with a temperature above 550 °C showed superparamagnetic behavior. Within the different calcination temperatures, ZnFe-MMO synthesized at 650 and 850 °C showed a similar M_S_ value (ca. 40 emu g^−1^), which was higher than the M_S_ value of Zn_2_Fe-MMO-750 being 26 emu g^−1^. Next, to investigate the optimal calcination temperature for MMO as an MPI tracer, the MPI measurement was conducted on Zn_2_Fe-MMO annealing at increasing temperatures. The two-dimensional projection of MPI and the line MPI spectrum (Figure 3B) showed that Zn_2_Fe-MMO-650 had a stronger contrast ability than other MMO samples. Zn_2_Fe-MMO-550 did not show an MPI performance, which was attributed to no superparamagnetic properties of Zn_2_Fe-MMO-550. MPI point-of-spread function spectra of the MMO nanoparticles showed the following MPI signal intensity: Zn_2_Fe-MMO-650 > Zn_2_Fe-MMO-850 > Zn_2_Fe-MMO-750 > Zn_2_Fe-MMO-550 (Figure 3D). The Zn_2_Fe-MMO-850 nanoparticles showed a lower MPI intensity, but a similar M_S_ value compared to Zn_2_Fe-MMO-650, likely due to the destroyed morphology and aggregated status of Zn_2_Fe-MMO-850, which declined the Brownian motion of nanoparticles in solution. All these results demonstrated that MMO nanoparticles at the designed ratio of Zn/Fe = 2:1 and calcination temperature of 650 °C have an optimal magnetism and MPI performance and were selected to be used in the following studies.

### 3.3. Chemical Composition, MPI Performance, and Cytotoxicity of Zn_2_Fe-MMO-650

**Figure 4:**
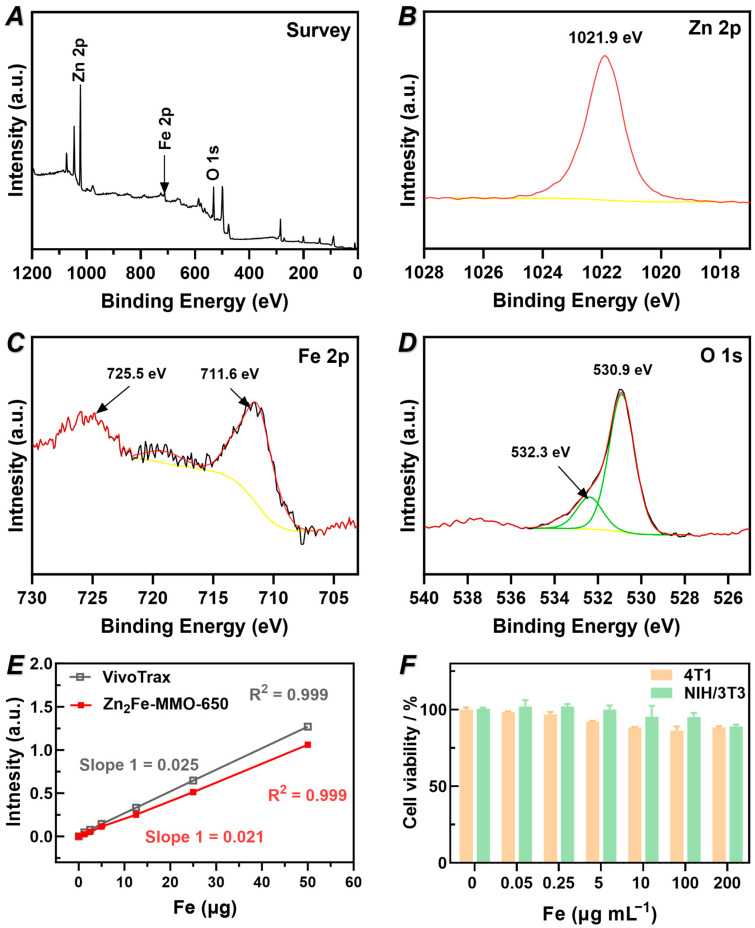
XPS spectra of Zn_2_Fe-MMO, (**A**) survey, (**B**) Zn 2p, (**C**) Fe 2p, and (**D**) O 1s. Yellow line represents background, red line represents fitting curve using Avantage software, black line represents original data, and green line represents split peaks. (**E**) Plot of Zn_2_Fe-MMO-650 signal intensity relative to commercial VivoTrax as a function of Fe mass. (**F**) Cytotoxicity after 24 h incubation with Zn_2_Fe-MMO-650 nanoparticles over a range of concentrations on 4T1 and NIH/3T3 cells.

The chemical states and composition of the as-prepared Zn_2_Fe-MMO-650 powders were measured by X-ray photoelectron spectroscopy (XPS). The survey spectrum reveals that the powders were composed of Zn, Fe, and O (Figure 4A). The high-resolution XPS analysis of Zn, Fe, and O was further conducted. As depicted in the Zn binding energy spectrum, a peak centered at 1021.9 eV corresponded to the binding energy of Zn 2p_3/2_, indicating the oxidation state of Zn^2+^ in the sample (Figure 4B) [30]. In Figure 4C, the signal of Fe 2p of the Fe^3+^ peaks convoluted by Fe 2p3/2 (711.6 eV) and Fe 2p1/2 (725.5 eV) refer to a small positive shift [31,32]. The high-resolution XPS spectrum of O 1s could be resolved into two peaks at 530.9 and 532.3 eV, which were attributed to the lattice oxygen binding with Fe and Zn of Zn_2_Fe-MMO-650 (Figure 4D) [33].

**Figure 5:**
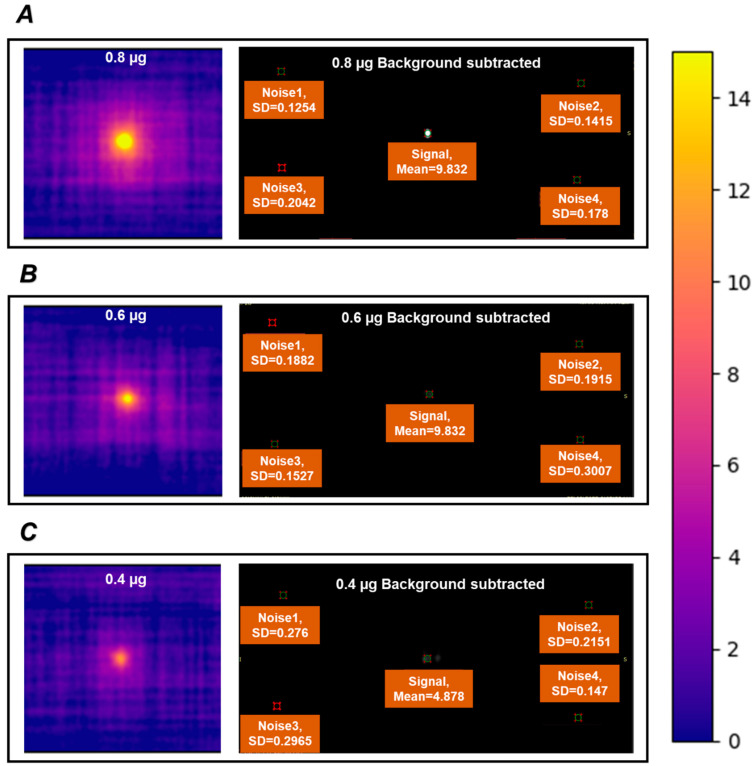
Signal-to-noise ratio of Zn_2_Fe-MMO-650 in an Fe mass of (**A**) 0.8 µg, (**B**) 0.6 µg, and (**C**) 0.4 µg.

To study the MPI signal linearity of Zn_2_Fe-MMO-650, a series of 2D projections was acquired with samples of different Fe masses. The MPI signals of Zn_2_Fe-MMO-650 showed a highly linear response to the Fe dose over a broad range concentration of Fe (R^2^ = 0.999, Figure 4E), indicating the potential to facilitate quantitative image analysis. It is worth noting that the MPI signal of Zn_2_Fe-MMO-650 was very close to that of the commercial MPI tracer VivoTrax (83% signal of VivoTrax), endowing the MMO potential for in vivo imaging applications (e.g., catheter steering and stem cell imaging) [34,35,36]. To obtain an indication of the sensitivity of our tracer in the MPI scanner, samples with a low concentration of Fe mass (0.4 to 0.8 µg of Fe) were scanned in the PCR tubes and compared to the signal background. The Zn_2_Fe-MMO-650 nanoparticles exhibited a signal-to-noise ratio of 60.6, 38.8, and 20.9 in the Fe mass of 0.8, 0.6, and 0.4 µg after subtraction of the background signal (Figure 5A–C), demonstrating that Zn_2_Fe-MMO-650 has the sensitivity to detect the MPI signal at a very low iron mass of 0.4 µg. The signal-to-noise ratio was calculated by the mean signal of the same region of interest (ROI) in each sample divided by the standard deviation within the same ROI in four corners determined as noise (Figure 5A–C). Next, the spatial resolution of Zn_2_Fe-MMO-650 for MPI was measured using a resolution phantom. Two microbore tubes were filled with 0.5 µL of the nanoparticles and were parallelly arranged in a linear array with an edge-to-edge distance of 3.8 mm between these two tubes (Figure S6A–C), showing that the signal can still be detected in a very close distance compared to the reported 3.1 mm distance [37]. The in vitro biosafety of Zn_2_Fe-MMO-650 was examined by measuring the cytotoxicity of cells compared with the untreated cells via a CCK-8 assay. Excellent cell viability (>85%) was observed on both the 4T1 breast cancer cells and NIH/3T3 fibroblast cells at an iron concentration as high as 200 μg mL^−1^ (Figure 4F).

### 3.4. In Vivo MP Imaging Evaluation

**Figure 6:**
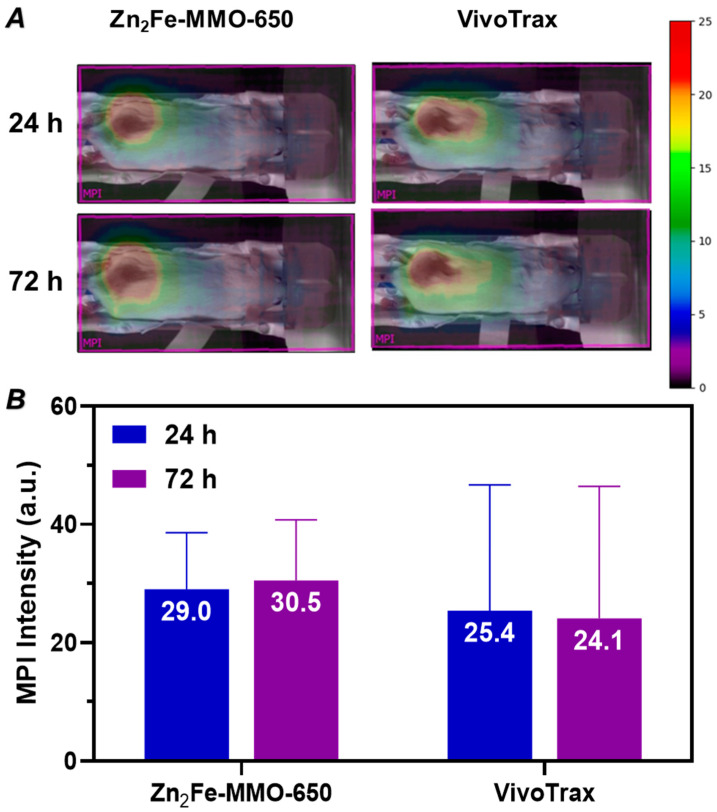
(**A**) Two-dimensional projection MPI images of mice bearing 4T1 subcutaneous breast tumors that were injected i.t. with 40 µL of Zn_2_Fe-MMO-650 (145 µg Fe mL^−1^) or VivoTrax (145 µg Fe mL^−1^) nanoparticles at 24 and 72 h post-injection. (**B**) Quantification of net MPI signals of tumor areas shown in (**A**).

Encouraged by the desirable in vitro MPI performance and high biosafety of Zn_2_Fe-MMO-650, the in vivo MPI performance of nanoparticles was further assessed by an intratumoral administration of Zn_2_Fe-MMO-650 in 4T1 tumor-xenografted BALB/c mice. The animal work was performed with the approval of the Animal Ethics Committees of the University of New South Wales. The commercial MPI tracer VivoTrax of an equivalent Fe dose (40 µL at 145 µg Fe mL^−1^) was used as a control. After an injection of Zn_2_Fe-MMO-650 or VivoTrax, mice were scanned using 2D projection MPI at different time intervals (24 and 72 h). Strong MPI signals could be detected from the tumor area without signals from surrounding anatomy in both the MMO- and VivoTrax-treated groups in Figure 6A. An MPI signal analysis showed that the signal intensity is comparable between the MMO and VivoTrax groups at each time point (Figure 6B). Despite the uncertainty represented by the large error bars, the net average MPI signal in the MMO group is slightly higher than that in the VivoTrax group. Particularly at 72 h post-injection, the MMO group demonstrated a higher mean MPI signal (1.27-fold) compared to the commercial tracer VivoTrax. These results indicate that Zn_2_Fe-MMO-650 tends to have longer retention times in the tumor and may have the potential for prolonged tumor studies in vivo to quantify nanoparticles in tumors by MPI.

## 4. Conclusions

We designed and synthesized Zn/Fe mixed metal oxide nanoparticles from LDH synthesized in water followed by calcination, which demonstrated the potential and suitability of Zn/Fe mixed metal oxide nanoparticles as MPI tracers. The Zn/Fe molar ratios and calcination temperatures of the nanoparticles were investigated and optimized as the two key parameters which affect the magnetic properties of particles and their MPI performance. The optimal MPI performance was found in Zn_2_Fe-MMO-650, which demonstrated desirable biocompatibility in in vitro cell study. Using an in vivo 4T1 tumor-bearing mouse model, the intratumoral administration of Zn_2_Fe-MMO-650 demonstrated comparable MPI signals in comparison to commercial VivoTrax. This proof-of-concept study indicated the potential of using MMO as an MPI tracer. It could be expected that stabilizers and cancer-specific ligands can be grafted to the nanoparticles and evaluated in a tumor cell-labeled animal model to further study the MPI performance of Zn_2_Fe-MMO-650 nanoparticles.

## Data Availability

Data will be made available on request.

## Supplementary Materials

The following supporting information can be downloaded at https://www.mdpi.com/article/10.3390/nano14231964/s1: Figure S1. PXRD patterns of LDH at Zn/Fe molar ratios of 2:1, 3:1, and 4:1; Table S1. ICP analysis results of Zn/Fe molar ratios in ZnFe-LDH and corresponding ZnFe-MMO samples; Figure S2. FTIR spectra of LDH at Zn/Fe molar ratios of 2:1, 3:1, and 4:1; Figure S3. FTIR spectra of ZnFe-MMO at Zn/Fe molar ratios of 2:1, 3:1, and 4:1; Figure S4. TEM images of (A) Zn_2_Fe-MMO-550, (B) Zn_2_Fe-MMO-750, and (C) Zn_2_Fe-MMO-850 (scale bar = 200 nm); Figure S5. FTIR spectra of Zn_2_Fe-MMO at calcination temperatures of 550 °C, 650 °C, and 850 °C; Figure S6. The spatial resolution study of Zn_2_Fe-MMO-650 shown by (A) photograph, (B) two-dimensional MPI image, and (C) linear scanning MPI spectrum.
