# Supplementary material for: Engineering Zn/Fe Mixed Metal Oxides with Tunable Structural and Magnetic Properties for Magnetic Particle Imaging
Source: Nanomaterials (Basel). 2024 Dec 7;14(23):1964. doi: 10.3390/nano14231964 (PMC11643550; doi:10.3390/nano14231964)
Supplement: Supplementary file 1 [file nanomaterials-14-01964-s001.zip › nanomaterials-3323600-supplementary.pdf]

Supplementary information

Engineering Zn/Fe mixed metal oxides with tunable structural and magnetic properties for magnetic particle imaging

Qianyi Zhang, Bing Sun, Saeed Shanehsazzadeh, Andre Bongers, Zi Gu*

Dr. Q. Y. Zhang, Dr. B. Sun, Dr. Z. Gu

School of Chemical Engineering, University of New South Wales, Sydney, NSW 2052, Australia

E-mails: zi.gu1@unsw.edu.au

Dr. Q. Y. Zhang

School of Chemistry, University of Sydney, Sydney, NSW 2006, Australia

Dr. A. Bongers, Dr S. Shanehsazzadeh

Biological Resources Imaging Laboratory, Mark wainwright Analytical Centre, The University of New South Wales, Sydney

Dr. Z. Gu

Australian Centre for NanoMedicine (ACN), University of New South Wales, Sydney, NSW 2052, Australia

UNSW RNA Institute, University of New South Wales, Sydney, NSW 2052, Australia

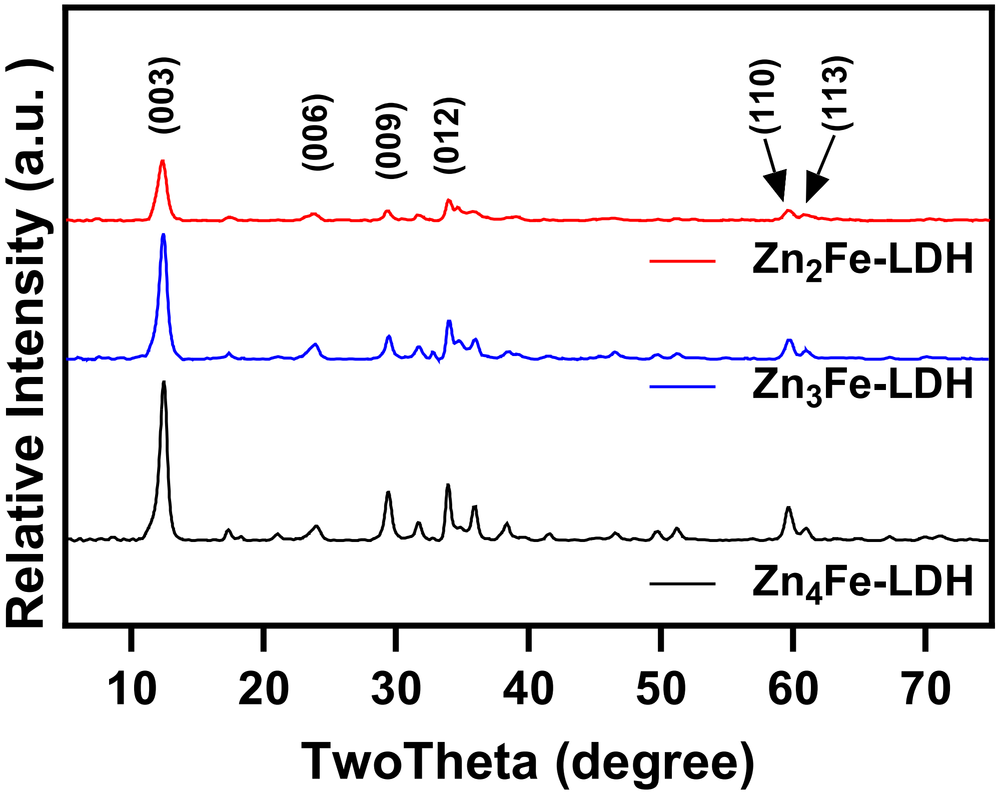

Figure S1 PXRD patterns of LDH at Zn/Fe molar ratios of 2:1, 3:1, 4:1.

Table S1 ICP analysis results of Zn/Fe molar ratios in ZnFe-LDH and corresponding ZnFe-MMO samples.

	**Designed Zn/Fe molar ratio**	**Actual Zn/Fe molar ratio**
**ZnFe-LDH**	2:1	1.5:1
	3:1	2.4:1
	4:1	3.4:1
**ZnFe-LDO**	2:1	1.5:1
	3:1	2.4:1
	4:1	3.1:1

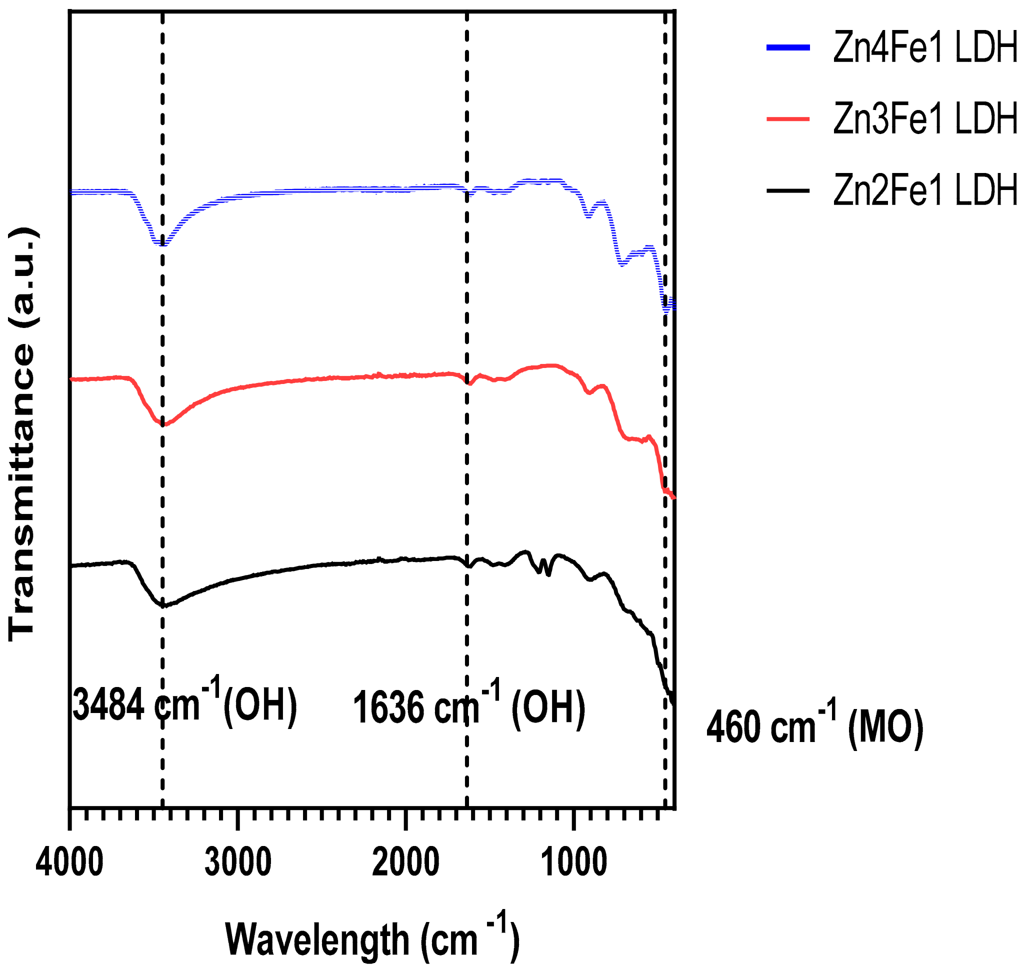

Figure S2 FTIR spectra of LDH at Zn/Fe molar ratios of 2:1, 3:1, 4:1.

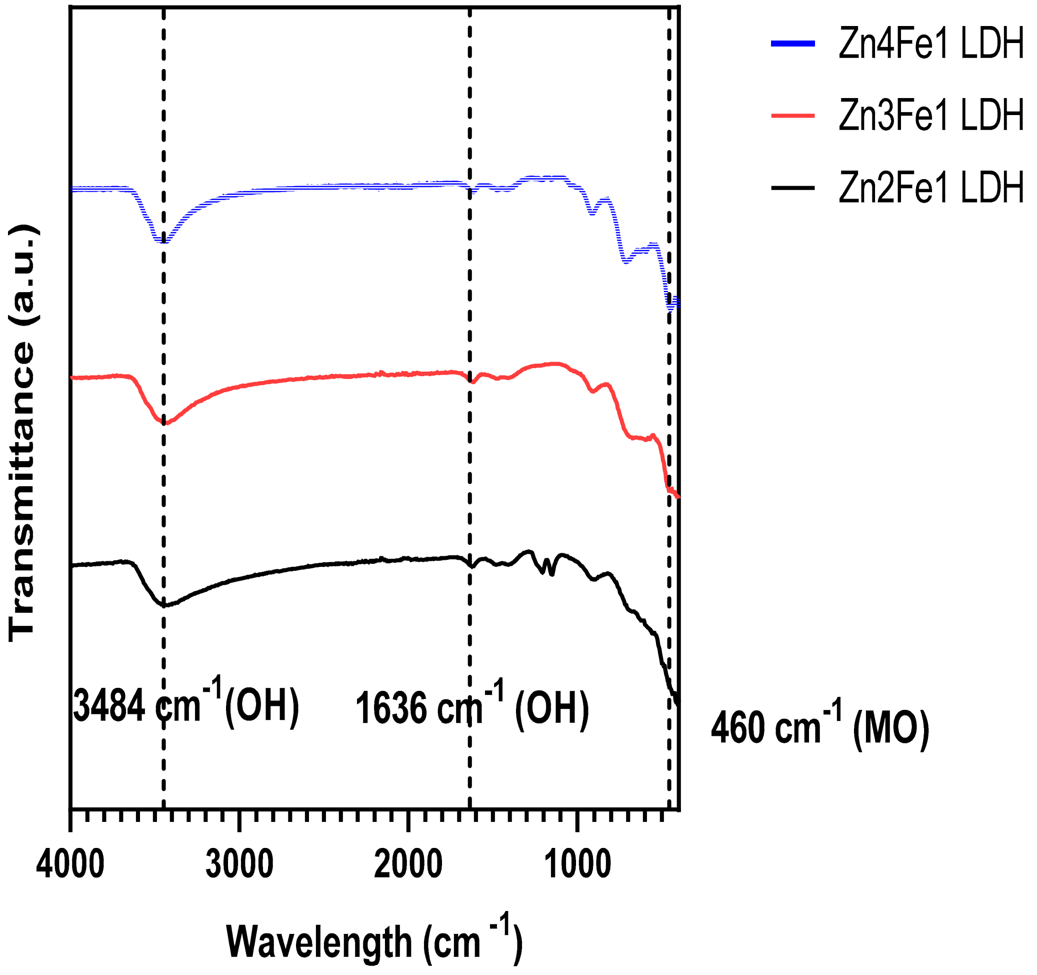

Figure S3 FTIR spectra of ZnFe-MMO at Zn/Fe molar ratios of 2:1, 3:1, 4:1.

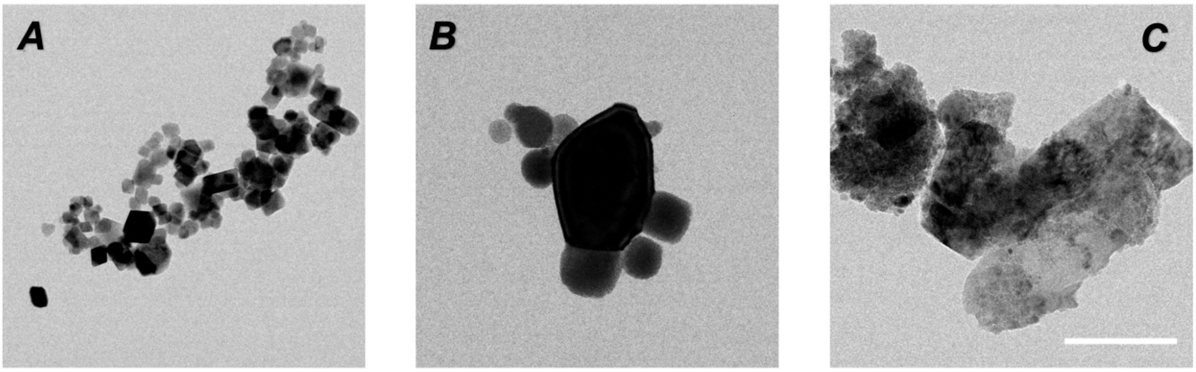

Figure S4 TEM images of (A) Zn_2Fe-MMO-550, (B) Zn_2Fe-MMO-750, (C) Zn_2Fe-MMO-850 (scale bar = 200 nm).

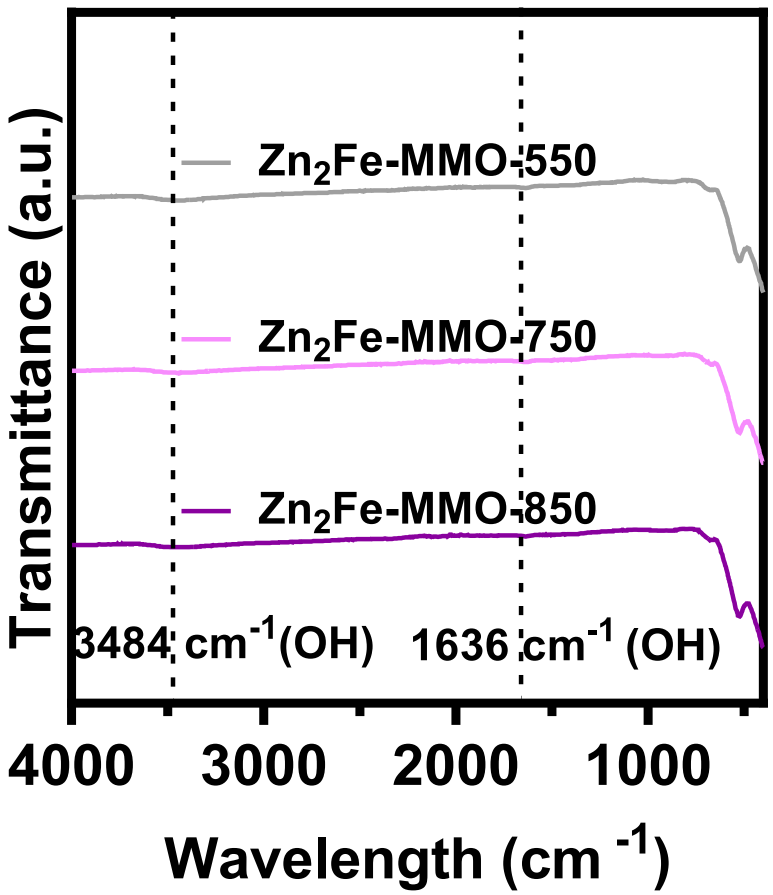

Figure S5 FTIR spectra of Zn_2Fe-MMO at calcination temperatures of 550 °C, 650 °C, and 850 °C.

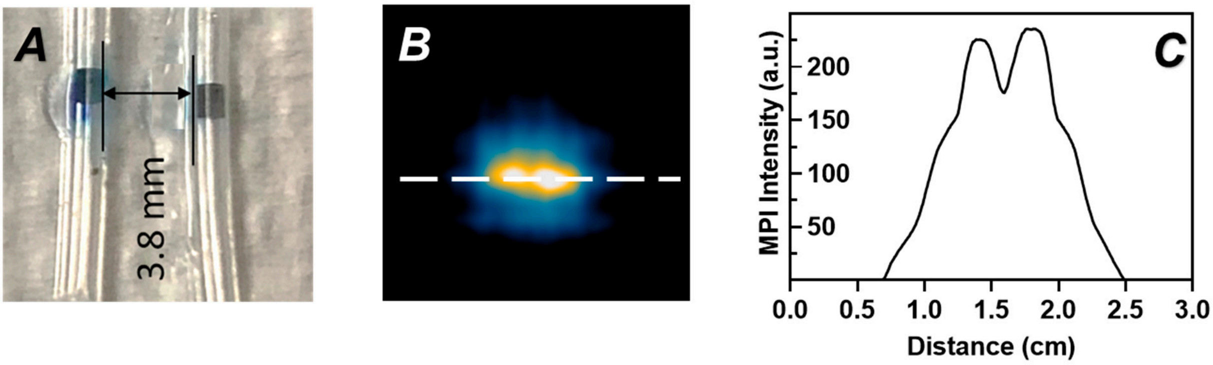

Figure S6 The spatial resolution study of Zn_2Fe-MMO-650 shown by (A) photograph, (B) two-dimensional MPI image, and (C) linear scanning MPI spectrum.
